# Grand challenges in space synthetic biology

**DOI:** 10.1098/rsif.2015.0803

**Published:** 2015-12-06

**Authors:** Amor A. Menezes, Michael G. Montague, John Cumbers, John A. Hogan, Adam P. Arkin

**Affiliations:** 1 California Institute for Quantitative Biosciences, University of California, 2151 Berkeley Way, Berkeley, CA 94704-5230, USA; 2 Applications of Vital Knowledge, 113 Chestnut Hill Way, Frederick, MD 21702, USA; 3 NASA Ames Space Portal, NASA Ames Research Center, MS 555-2, Moffett Field, CA 94035, USA; 4 Bioengineering Branch, NASA Ames Research Center, MS 239-15, Moffett Field, CA 94035, USA; 5 E.O. Lawrence Berkeley National Laboratory, 1 Cyclotron Road, MS955-512 L, Berkeley, CA 94720, USA; 6 Department of Bioengineering, University of California, Berkeley, CA 94720, USA

**Keywords:** resource utilization, manufacturing, life support, space medicine, space cybernetics, terraforming

## Abstract

Space synthetic biology is a branch of biotechnology dedicated to engineering biological systems for space exploration, industry and science. There is significant public and private interest in designing robust and reliable organisms that can assist on long-duration astronaut missions. Recent work has also demonstrated that such synthetic biology is a feasible payload minimization and life support approach as well. This article identifies the challenges and opportunities that lie ahead in the field of space synthetic biology, while highlighting relevant progress. It also outlines anticipated broader benefits from this field, because space engineering advances will drive technological innovation on Earth.

## 1. Introduction

The field of space synthetic biology, which lies at the intersection of aerospace engineering and bioengineering, holds great promise for long-duration space missions: for instance, synthetic biology approaches can transform both astronaut waste resources and *in situ* destination planet resources into practical products while consisting of less mass (saving as much as 26–85% depending on the application) than conventional abiotic means [1]. Biological technologies can also lower power demand and launch volume, two other important space metrics, by innately harnessing solar energy and by growing only upon activation using available destination nutrients, respectively. In addition to cost-effectiveness, these technologies provide an alternative means of realizing mission objectives that constitute redundant mechanisms over traditional abiotic approaches, thereby improving astronaut safety. Moreover, biological technologies are versatile and vast. Microbes that can be harnessed for space use come from all three biological domains, namely bacteria, archaea and eukaryotes. These organisms are feasible lightweight tools that not only represent exceptionally viable chassis for space application [2–5], but also represent an expansion into new biological kingdoms in contrast to current space technologies that, when incorporating biology, have only considered plants (e.g. for food).

**Figure 1.**
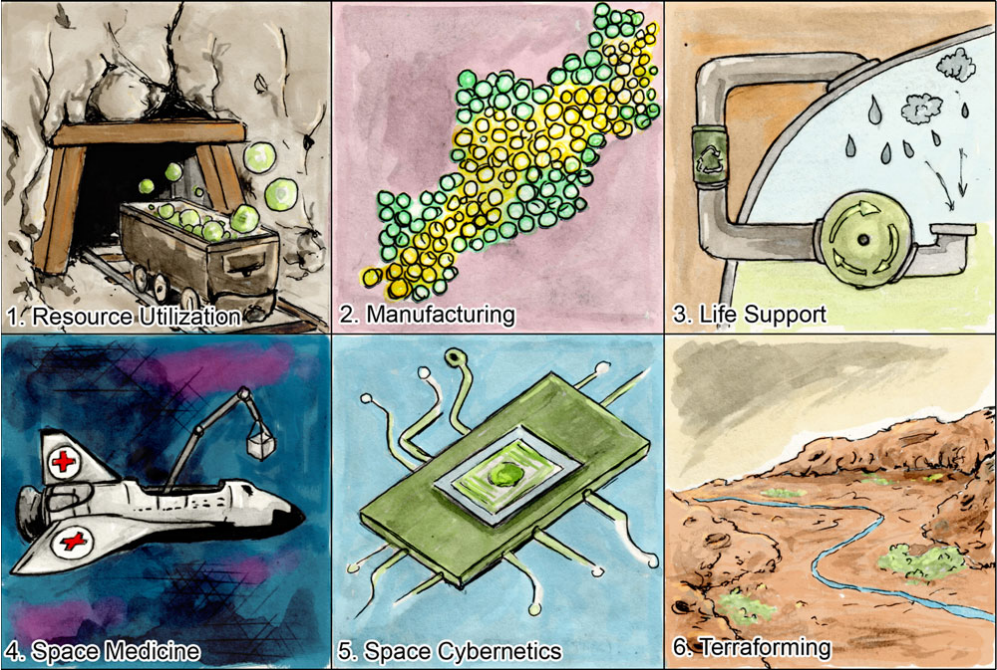
The six grand challenges of space synthetic biology (art by Hugo Teixeira).

Accordingly, there is a need to identify the potential near-term and longer-term goals that space synthetic biology can progress towards. There is also a need to outline the anticipated techniques that can achieve these objectives, and a need to document the impact that attaining these milestones can have on the space community and, more broadly, humankind. The associated challenges and opportunities deal with the biological extraction and utilization of limited space resources, the manufacture and construction of products useful in space, the support of human life, the treatment of human health, the development of biological devices that can emulate and interact with non-biological components and, ultimately, the large-scale transformation of worlds from harsh environments into more hospitable ones. These challenges and opportunities are illustrated in figure 1, summarized in box 1, and elucidated in the following sections.

Box 1. **Summary of the grand challenges in space synthetic biology.**

1. Resource utilization

 (a) Ensuring functionality in extreme environments.

 (b) Providing the capacity to harness three kinds of resources: wastes, volatiles and minerals.

 (c) Producing feedstocks for manufacturing processes and cell-based biomaterials for construction processes.

2. Manufacturing

 (a) Satisfying construction-related desires with adhesives to bind regolith, biocement and biopolymers.

 (b) Generating fuel for power and propulsion.

 (c) Revisiting abiotic manufacturing and construction technologies to leverage existing or synthetic biology capabilities.

3. Life support

 (a) Improving the biological management of waste, especially wastewater.

 (b) Treating, conditioning and recycling air, water and solid wastes through incorporating biology into traditionally inanimate structures, e.g. creating a ‘living’ habitat.

 (c) Producing flavourful, texture-rich and nutritious food.

 (d) Providing nutrients, and assisting with the recycling of nutrients.

4. Space medicine and human health

 (a) Preventing disease and maintaining the human microbiome.

 (b) Manufacturing synthetic drugs to combat disease, radiation damage and the effects of reduced gravity.

 (c) Developing radiation-resistant, self-healing protective clothing and personal shielding.

5. Space cybernetics

 (a) Developing device-level biological control systems: biological sensors, actuators and controllers.

 (b) Designing biological control systems that are either completely composed of biological parts, or that partially integrate biological controllers and systems with abiotic sensors and actuators as a form of artificial life.

6. Terraforming

 (a) Paraterraforming with few multi-functional species that complete the carbon and nitrogen cycles.

## 2. Challenge 1: Resource utilization

*In situ* resource utilization (ISRU) obviates the expensive transport of equipment and consumables from the Earth into space. Biological techniques represent a new and productive ISRU approach [1,6]. The first subchallenge that this new approach must overcome is adapting life to extreme environments (e.g. by advancing preliminary efforts [7,8]) to ensure the reliability of microorganism performance in bioreactors that experience large swings in temperature, ionizing radiation, and minimal nutrient and oxygen availability. This engineering will also determine the extent to which additional bioreactor protection is required to reduce the effects of environmental extremes on the microorganisms. For plants, ongoing terrestrial efforts to impart drought-resistance [9] are very relevant here, given a scarcity of extraterrestrial water.

Second, future biological processing technology (either anaerobic or aerobic, depending on oxygen availability) must be developed for each of three kinds of space resources: solid waste (available on manned spacecraft), volatiles (variably available by composition from life support systems and also from some asteroids, subsurface lunar regolith and planetary atmospheres) and minerals and other geological materials (available from asteroids, moons and planetary surfaces). Solid waste refers to metabolic human waste as well as packaging materials and trash from experiments and crew activities that can yield carbon via pyrolysis. Earth-based anaerobic sewage treatment [10] and composting [11] are intrinsically amenable to synthetic biology improvement, and have recently been shown capable of energy recovery through the production of a known space fuel, nitrous oxide [12]. Volatiles such as carbon dioxide and nitrogen have significant biomanufacturing utility that can be furthered by synthetic biology (see [1]), and progress towards a single platform (perhaps consisting of synthetic cyanobacteria [13]) for handling multiple volatiles is also desirable.

Enhancing biomining and bioleaching [14–17] for asteroid and planetary deployment is another space synthetic biology opportunity. Instead of employing traditional ore extraction and smelting equipment, space resources may be harvested by either removing a matrix of surrounding rock in bioreactors using acid-producing microbes that target only the matrix, or by removing the resource from the matrix using bacteria that perform redox biochemistry to dissolve the resource, which is then recovered by electroplating. Associated subchallenges include chemical specificity in the mobilized metals, and complications arising from the composition of the matrix (e.g. the presence of heavy metal toxins).

Yet other subchallenges here are the production of intermediate resources (i.e. feedstocks) for downstream (possibly biological) space manufacturing processes, and the production of cell-based biomaterials for construction. Such biomaterials have lower production mass requirements [1] and an innate embedded biological control ability (challenge 5) that bestows desirable properties such as self-healing and the memory of several shapes. Menezes *et al.* [1] advocated microorganisms that generate and use acetate and methane intermediates, given their known production efficiency, and also suggested polyhydroxyalkanoate biopolymers for use in three-dimensional printing-based construction. The set of suitable and cost-effective resource intermediates is still relatively unexplored, with individual feasibility dependent on post-synthetic biology volumetric yields and efficiency.

This grand challenge is the most fundamental: it literally provides building blocks for manufacturing (challenge 2), it supplies the materials not fully recovered by regenerative life support systems (challenge 3), and it is a stepping-stone towards paraterraforming (challenge 6). Solving this challenge helps reduce the limited-resource problem facing space synthetic biology that constrains feasible inputs for downstream processes. Back on Earth, this challenge has direct implications on energy production and mining technology.

## 3. Challenge 2: Manufacturing

The manufacturing grand challenge focuses on biological and non-biological outputs that can be generated from inputs resulting from solutions to challenge 1. Although such outputs also cater to more traditional basic needs such as water, air, food and clothing, the discussion of such outputs is deferred to the life support and healthcare grand challenges (numbers 3 and 4, respectively). Thus, the considered outputs here are associated with shelter, astronaut comfort and, eventually, industry and economy for one or more extraterrestrial colonies and Earth. The most pressing near-term subchallenge consists of using biology to make bricks or building materials by binding regolith together, perhaps by adapting natural adhesives such as mussel foot protein [18,19] for space, or by using microbes to precipitate calcium and/or iron from regolith to make biocement, construction biopolymers, etc. [20,21]. A synthetic programmed pattern formation process developed in response to challenge 5 can leverage these building materials and assist with habitat/furniture construction: microbial cells can be controlled to line up their secretions, or form layers to build up a structure, and so on. Another sample desirable manufacturing output is a hydrocarbon fuel such as methane [1] or a more conventional fuel such as hydrazine [22] that can power a colony and be used for propulsion. It is also possible for abiotic manufacturing techniques to use synthetic biological outputs; examples include three-dimensional printers that use biopolymers [1], radiation-shielding tiles that are made from biologically solidified surfaces [23], self-replicating factories [24] (making solar cells on the lunar surface for instance) that harness biological processes because of built-in self-replication technology, etc.

## 4. Challenge 3: Life support

Previous efforts such as NASA's controlled ecological life support system and the European Space Agency's MELiSSA [25] programmes to incorporate biology into life support systems have often focused on developing complex, large-scale ‘closed ecosystems’ to replicate the functions of physico-chemical life support systems such as the International Space Station's ECLSS [26], while also providing food. Although synthetic biology can assist in such efforts (through a cyanobacteria-based life support system [27] for example), it will excel through applying specific synthetic biology techniques to improve the individual modules of required life support systems. For instance, synthetic biology can assist with wastewater treatment in at least two ways: in microbial fuel cells that rapidly, efficiently and robustly remove organics, nitrogen and phosphorus and also generate electricity [28], and in converting wastes into compounds that have food, therapeutic and chemical applications [29,30]. Another life support subchallenge is how to incorporate synthetic biology organisms directly into (the walls of) a habitat, to recycle carbon dioxide into breathable oxygen and provide a secondary layer of radiation protection that is self-healing [31]. Other subchallenges include how to generate nutrient-dense biomass that supplements astronaut dry-food while being versatile in flavour and texture [1,13], and how to recycle, convert and/or provide nutrients for downstream biological and bioreactor processors.

## 5. Challenge 4: Space medicine and human health

Disease prevention, disease cure and radiation protection are the most pressing subchallenges of the fourth grand challenge, closely aligning with current Earth-based medical synthetic biology research priorities [32,33]. Space agencies are also interested in other medical synthetic biology endeavours on microbiome maintenance and regulation [34,35]. Efforts to battle cancer tumours using synthetic biology [36,37] are particularly important in the light of the substantial space radiation that astronauts are exposed to on long-duration missions, coupled with their lack of access to traditional care because of travel distance and the mass of treatment equipment. To this end, the development of personalized approaches [38] that can monitor for and diagnose medical conditions, and also administer tailored treatments (e.g. melanin [39] and granulocyte-colony stimulating factor to counteract radiation [40] just as it does for chemotherapy) is imperative, and has obvious corresponding applications back on Earth in resource poor settings, for the military, for sun protection, etc. Space radiation also induces accelerated pharmaceutical expiry [41], and synthetic biology pharmaceutical development [1,42] that can allow astronauts to produce needed drugs almost in real time without massive chemical apparatus is another vital space technology. To prevent microbial cell ‘expiry’, storage in a small lead-lined container while inactive is a feasible means of supplementing a microorganism's natural ability to withstand the harsh rigours of space, as when protected by rock [4]. Because of the extremely small volume of a microbial colony, transporting the mass of such a shielding system is much more feasible than it would be for macroscopic organisms or abiotic systems. In addition, millions of bacteria can be just as easily transported on a space mission as a single bacterium; coupled with quality control of astronaut-activated bacteria via portable gene sequencers or reporter-gene systems, the extreme redundancy of transporting millions of bacteria per colony mitigates any risk of mutation away from the intended genotype owing to cosmic or solar radiation. Alternatively, plant synthetic biology [43,44] can also be used to produce necessary pharmacological compounds [45].

## 6. Challenge 5: Space cybernetics

**Figure 2.**
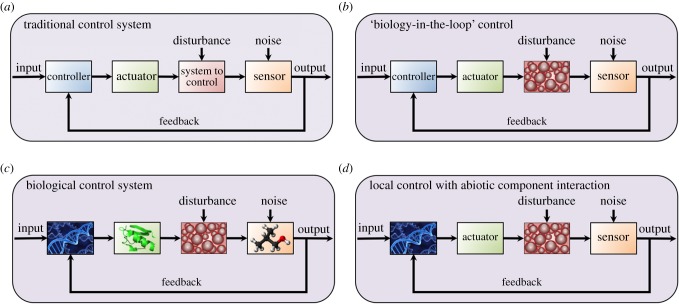
Synthetic biological control systems for use in space. (*a*) A traditional feedback control system consists of a controller, an actuator, a sensor and a system to be controlled, all arranged within a feedback loop. (*b*) ‘Biology-in-the-loop’ control refers to contemporary electromechanical (e.g. microfluidic or optical) techniques of externally controlling a biological system. (*c*) Challenge 5 moves towards a methodology that completely integrates biological controllers (perhaps based on gene regulatory networks), actuators (perhaps one or more proteins) and sensors (perhaps levels of chemicals of interest) with the biological system to be controlled (the control subchallenge). (*d*) Challenge 5 also includes the case where biological controllers and the systems to be controlled constitute separate biological subsystems that individually interact with abiotic sensors and actuators, all of which are part of a larger system, e.g. a hybrid robot (the artificial life subchallenge).

This systems engineering challenge will enable the construction and operation of robust and reliable space synthetic biology devices, surpassing direct application of microfluidics in a space environment [46]. The traditional control engineering approach to systems engineering (figure 2*a*) uses (possibly noisy) sensors to determine the operational output of the system to be controlled; a means of feeding back and communicating output signals and a similar means of transmitting desired inputs; and a controller to compare inputs and outputs and to actuate the system to ensure suitable performance while compensating for disturbances. One subchallenge here is therefore the creation of analogous biological equivalents. Examples include biosensors on spaceships and planetary habitats to accomplish environmental or radiation monitoring and to indicate the need for damage repair (perhaps using plant sentinels [47]); feedback and communication through biomembranes or between cells or even with local transceivers capable of interplanetary information transfer [48]; biological control elements that act locally to attain optimal behaviour or programmed pattern formation (for instance, to construct an arching habitat from layered cell secretions); and designing integrated biological controllers and systems in a way that mitigates unwanted module-interaction effects.

The above-described control subchallenge goes beyond ‘biology-in-the-loop’ control of the sort in figure 2*b* and [49,50], where electromechanical control is applied external to a host using feedback provided by internal biological sensor components. This control subchallenge (figure 2*c*) is related to another one that is schematically illustrated in figure 2*d*: that of space artificial life, where sentinel or drug-delivery hybrid robots are created from both biological and abiotic components. These components may interact via the mechanism in [51], for example. At a higher level, an artificial space self-reproducing system should emulate the resilience of a natural system to disturbances (meteoroid strikes, solar flares, etc.), and it should also efficiently search among its possible configurations in the aftermath of a disturbance to rebound more effectively. Such desirable behaviour could be achieved through a version of selection (e.g. directed evolution), and yet another subchallenge is enforcing longer-term control when designing space hybrid robots.

The broader impacts of the control subchallenge help solve open problems in industrial production and metabolic engineering. These include, respectively, the sensing of and (optimal) response to complex environments such as those found in large bioreactors, and providing a means of flux regulation to facilitate unnatural chemical production. Similar to the versatile abiotic applications of control engineering, it is anticipated that the accomplishment of generic biological control that is independent of host system and that can compensate for certain off-pathway effects, recoverable mutations and environmental fluctuations will have many uses beyond space-related applications. Additionally, solutions to the artificial life subchallenge may constitute future Earth-based medical technologies, for instance, hybrid robot versions of tumour-killing bacteria [36].

## 7. Challenge 6: Terraforming

Terraforming refers to the rendering of a non-terrestrial body into one capable of supporting Earth life, and is classified into two types: ‘true-terraforming’, which aims to establish an entirely self-contained, materially (but not energetically) self-sustained, self-regulated habitable environment equivalent to the biosphere of Earth, and ‘paraterraforming’, which retains the requirement that the environment be self-regulating and materially self-sustaining, but not that it be self-contained [52]. Because of the massive size of reasonable targets for true-terraforming (entire planet surfaces), true-terraforming is expected take centuries [53] and is outside the scope of this article. In paraterraforming, an enclosed environment materially separates the interior from the exterior, and maintains a stable habitable environment in the interior of the enclosure. Asteroids, localized regions of moon or planetary surfaces protected by domes, or caverns under these surfaces are all potential paraterraforming sites. Paraterraforming is a task that can be pursued on a reasonable time scale with massively fewer resources than true-terraforming.

Synthetic biology provides the opportunity to improve upon a past paraterraforming trial, the Biosphere 2 project [54,55], which attempted to create a materially self-enclosed system that was capable of supporting eight humans for two years. The project discovered that its fertile carbon-rich soils acted together with the microbial metabolism to bind oxygen in carbon dioxide, which was in turn absorbed by calcium in the structure's concrete walls [56]. The project also found that its initial biodiversity of multiple semi-separated ecosystems with hundreds of species (including pollinating insects) was lost over time. The project's design further imposed caloric and nutrient restrictions on the Biosphere 2 crew [57]. Synthetic biology now affords the ability to tightly regulate a limited number of variables (e.g. by combining advances in challenges 3–5).

Simultaneous efforts in planetary protection [58] are paramount. This includes ensuring that organisms remain contained within an enclosure and are inactive beyond it, and that techniques be developed to intercept organism spread after accidental contamination. However, it should be pointed out that if paraterraforming is required in the first place, then by definition, the environment outside the paraterraformed enclosure is not suitable for terrestrial life, which makes issues of containment easily addressed.

Paraterraforming is perhaps a grander challenge than the others listed above, but the individual tasks required (composting, soil improvement, farming, remediation, etc.) are not unfamiliar ones, and functionally comprise a scaled-up version of a terrarium. Synthetic biology advances that address this challenge have the potential for correspondingly greater Earth benefits, ultimately leading to better local remediation efforts and perhaps better global climate-change mitigation techniques.

## 8. Summary and concluding remarks

Space synthetic biology holds great future promise as a new and exciting biotechnology field, with numerous directions for fruitful research that are grounded in technologies already in development today. These opportunities are summarized in box 1, and provide a glimpse of exciting possibilities that also have immense Earth benefit.
